# The Female Urethra: A Source of Trouble Often Overlooked in Our Gynæcological Investigations

**Published:** 1890-09

**Authors:** K. P. Moore

**Affiliations:** Macon, Georgia


VOL. VII. SEPTEMBER, 1890. NO. 7.

### Original Communications.

### THE FEMALE URETHRA: A SOURCE OF TROUBLE OFTEN OVERLOOKED IN OUR GYNÆCOLOGICAL INVESTIGATIONS.*

BY K. P. MOORE, M. D., MACON, GEORGIA.

Mrs. C., age about forty-eight, came to me from a neighboring town, some months ago, to be treated for some supposed uterine ailment. She had been married about twenty-five years, no children, and had not yet reached the menopause. For quite a number of years she had been a victim to much pain and suffering, and was now almost a complete physical and nervous wreck. Possessed of a fair proportion of this world's goods, she had sought relief in various and sundry ways and places. She had visited many noted springs and health resorts; had suffered many things of many doctors; had taken, perhaps, largely more than her share of legitimate pills and powders, to say nothing of the limitless number of nostrums, blood purifiers, liver

*Read before Georgia Medical Association at Brunswick, May 1890.

and kidney cures, uterine tonics, etc., *ad nauseam*. And yet, like the poor woman we read of in the old book, "neither could be healed of any."

She was rather above the average in culture, as well as good common sense, and being naturally possessed of a sanguine temperament and great will power, she was saved from falling a victim to the opium or some other miserable habit.

Some months previous to her coming to me she had been in an infirmary conducted by one of the best gynæcologists of our State. That she had at that time some uterine trouble, and that she was judiciously treated, is not at all questioned. She was kept under constant treatment there for several months, and received some benefit, and her general health was considerably built up and improved; and yet she was still an invalid and a great sufferer.

On an examination by the touch I found the vaginal canal considerably smaller than normal for a married woman, with a very considerable amount of vaginismus. The degree of vaginismus present made digital examination somewhat painful. I interrogated her liege-lord in reference to coition, and was informed that he had rarely ever enjoyed so intimate a relation with her for a number of years, on account of the extreme pain which it gave her.

The uterus was in its normal position, somewhat atrophied, and pretty freely movable, and while there was some irritability of this organ, yet I could not find sufficient disease of the uterus or ovaries to account for her condition. She had passed through the hands of some skillful gynæcologists, who I felt sure had given her the very best treatment, so far as treatment directed to the uterus should go.

A short while before this, I had fortunately had the pleasure of attending a number of old Dr. Emmett's clinics at the woman's hospital in New York. At one of these he did his button-hole operation, and dwelt at some length upon female urethral troubles, and their long train of nervous consequences. Having only so recently had my mind thus impressed, I naturally fell to investigating in this direction for an explanation of this patient's condition.

Hid away in the urethra, just a little way behind the meatus, I found what I feel almost sure was the true origin and constant source of this good lady's long years of patient suffering and broken health.

On the inferior wall, and just within the urethra, were two papillary angiomata, not larger than a very small pea. These growths were exceedingly sensitive to the touch, and would bleed upon manipulation. It required but very slight pulling open of the meatus to expose the nearest one to view, and indeed, while it was not actually in sight without opening the meatus, yet it was so near as to give a slight pouting to the meatus. The other was a little to one side of the urethra, and from a quarter to half an inch behind the first one. These insignificant little tumors had, on the principle of a fissure in ano, set up a train of reflex neuroses that had undermined this patient's health and constitution, and made shipwreck of her health, and life a miserable burden.

This case is reported to illustrate the force of the idea set forth in the title of this paper ; and Dr. Emmett was eminently correct when he laid at this door the origin and source of many female troubles. There can be no question but that we too often pass by this cave of concealment in our eager search into the deeper anatomical researches for the enemy of female health. Here he often lurks unseen and undiscovered, and thus having full and undisputed sway, plays sad havoc with many a nervous system ; and makes miserable wrecks not only of the poor women themselves, but often of the happy households over which they preside.

It is not my purpose to go into the *many* pathological conditions and changes to which the female urethra is subject. Indeed, in a short paper like this, I could not go at length into the discussion of *any* of them. Yet it is well in passing to mention that we *may* have to deal with such affections, for instance, as dilatation, culminating, it may be, in urethrocele, stricture, dislocations, prolapsus, urethritis, both specific and non-specific, ulceration, catarrh of Skene's glands, etc.; and while it is true that these conditions are not very frequent, yet we should not be unmindful of their *possible* existence in any case, and govern ourselves accordingly.

It is my purpose to speak more particularly of what may be termed *new* growths, and even among these their name may be called legion; for the *urethral* growths and tumors are similar in character and organization to analogous structures of other parts of the body, and subject to the same laws and causes; and hence the same varieties are found here as elsewhere in the human system. For instance, we may have urethral polypi, fungoid excrescences, fibroma, sarcoma, or epithelioma; but on account of the infrequency of their occurrence, and with no desire to lengthen my paper beyond proper bounds, I will not consume the valuable time of this presence in their discussion.

Varices, or urethral hemorrhoids, is another pathological condition, sometimes superinduced by prolapsus or other uterine displacement.

My object in the mention of these various possible conditions of the female urethra is to emphasize the importance of a thorough search into *every* case brought under our notice, even though there was no *very decided* symptom pointing in this direction. Narrowing this paper down, then, to the one most frequently encountered, as well, unfortunately, as the one which gives the most distressing results, viz.: papillary angioma, or caruncle, I propose to give some of the most prominent symptoms indicating its existence.

Except fissure in ano, there is probably no pathological condition of such apparent innocence and insignificance, capable of producing such a train of distressing and baneful consequences. These growths produce symptoms which bear no relation to their size. In the case reported in the beginning of this paper, the tumors were quite small and insignificant, compared to the general consequences which they produced. Nor were there any very emphatic or decided symptoms which were *purely* referable to the bladder or urethra. The *exquisite sensitiveness* and

*pain* on micturition, so often present in these cases, was comparatively absent in this.

Dr. William H. Baker, of Boston, in the American System of Gynæcology, says that, in his experience, there is no affection of the genito-urinary apparatus capable of producing such a total wreck of the nervous system, and such a condition of utter misery as urethral caruncle. He thus describes a typical case: "These growths are characterized by their exquisite sensitiveness to the touch and to the contact of urine. A caruncle no larger than a pin's head may cause the most intolerable agony with each act of micturition. This pain may be simply the result of friction of the stream against the tumor; but more often the distress is greatly augmented by a reflex vesical spasm, which renders micturition extremely difficult and painful, at the same time that its frequency is increased. The vaginal sphincter also participates in this reflex irritability, and a permanent vaginismus often renders coitus absolutely impossible. Walking, or even sitting, is painful to these patients, especially when the tumor protrudes more or less from the meatus. Hemorrhage from the growth is a prominent symptom in many cases, and a high grade of anæmia is often produced. Eventually the appetite begins to fail, the sleep is broken, and this, together with the constant worry and distress, and the not infrequent hemorrhage, may reduce the patient to a condition of physical and nervous exhaustion and debility such as is usually found only with serious constitutional disease."

Only a few days ago a patient came into my office with haggard looks, with pain and anguish delineated upon her face. It was not necessary to hear her tale of woe to be assured that she suffered. Her stomach had been made the receptacle of many a nauseous dose—copaiba, cubebs, turpentine, iron, the potashes, etc., had been given, and, of course, she was none the better for it all. I at once suspected urethral caruncle, and from the pain experienced at the mere touch of the vulva and meatus, I would almost have sworn to the diagnosis before seeing the urethra.

As before stated, the amount of pain and constitutional disturbance is not necessarily in proportion to the size of the caruncle. On account of their size and situation they often escape our attention, and we are apt to charge up the effect to some other cause. It is true that we often find very distressing symptoms referred to the urethra, when this organ is in a healthy state, or, at least, free from any organic lesion. We sometimes encounter cases where micturition is accompanied with considerable pain and tenesmus, especially so during the menstrual period, in which there is no organic change of the urethra, and the only explanation which we have to offer is that of reflex neurosis ; yet this fact should make us none the less careful in our investigations in any given case. A Jarvis speculum or an ordinary pair of dressing forceps will usually enable us to clear up the diagnosis. The favorite habitat of these growths is the immediate vicinity of the meatus, and usually on the floor of the urethra.

The object and scope of this paper would not allow any lengthy discussion of the differential diagnosis of the several new growths found in the female urethra. Indeed, so far as the *treatment* is concerned, it makes but little difference as to the *character* of the tumor, the indications are the same. As to the best remedies, in my judgment the knife stands at the head of the list. Excision, as complete as it is possible, is by far the most expeditious, as well as the most effective means of cure. Unfortunately the papillary angiomata, while it is the most distressing and most disastrous in its results, is also the most prone to return after treatment; hence the importance of making the removal as *complete* and *thorough* as possible. My rule has always been to make as clean and complete excision as possible, and then, in order to make assurance doubly sure, to cauterize the base or stump. The hyper-sensitiveness of these growths would certainly make the operation a painful one; yet in operating on at least a half dozen or more during the last year I have rarely found it necessary to administer an anæsthetic. I have always succeeded in producing sufficient local anæsthesia by cocaine. I introduce into the urethra a pledget of absorbent cotton saturated with a four per cent. solution of cocaine, and usually find at the end of fifteen or twenty minutes that the operation can be done with but little pain. The cotton is twisted loosely upon a sound, a tooth pick, match, or something of this sort. After it is sufficiently cocainized, I open the meatus and urethra sufficiently to bring the growth into view, and then seize it with a pair of bull-dog forceps, or if of much size and broad base, with a small vulsellum, and with curved scissors cut the whole thing out. Should the base be broad and flat, and any doubt be had as to the completeness of the removal, I should use the curette, and then if accessible, should use a Paquelin cautery, or what would be as effective, a red hot wire. In a tumor of so much vascularity we would naturally expect considerable hemorrhage, but I have never yet had any trouble in arresting hemorrhage by pressure, and the application of caustics. Should much trouble be encountered on account of hemorrhage, a large and closely fitting bougie or sound would arrest it, and this might be allowed to remain in the urethra for some hours if necessary. We should never lose sight of the fact that much harm may be done by lessening the urethral caliber in this operation, and while excision should be *complete* and *thorough*, in order to prevent a return of the growth, yet it would be an unfortunate result if we should superinduce a contracted urethra by the operation, a result almost or quite as bad in its consequences as the disease we seek to cure.

The subsequent management of the case would be such as would suggest itself to any thinking man. The vaginismus is to be overcome by dilatation, and the soothing sedative, local applications and douches. Of course the general condition of the patient should be looked after, and such constitutional treatment and management instituted as might from time to time be indicated.

There is one condition of which I wish to speak, and to which I have seen no reference in any of the literature to which I have had access. Indeed, my observation aud experience in this direction is too limited to offer the suggestion as a settled question, and yet it is quite a rational one, and I believe that further clinical observation will confirm my opinions. It seems to me that these neoplasms might be divided into irritable and non-irritable, or sensitive and non-sensitive growths.

I have referred in this paper only to the sensitive or irritable neoplasms and briefly mentioned the symptoms which their presence causes. And now I invite your attention to the other class of new growths, and which, on account of the absence of acute sensitiveness, are very liable to be overlooked in our gynæcological investigations. During the past several years, since I have had more gynæcological work to do than formerly, I have occasionally met cases of general decline, and a large amount of nervous irritability and pain, particularly of the hips, back and pelvic region generally, for which I could find no adequate explanation in the uterus or ovaries, and, upon making a more careful search, have found urethal neoplasm sufficiently large to lessen the caliber of the urethra, and set up a degree of local inflammatory condition; and yet, so far as these patients themselves knew, they had suffered but little if any inconvenience from their presence. On account of the absence of pain, I have denominated these growths "non-sensitive," and yet their existence is capable of producing a considerable amount of reflex nervous disturbances.

I mention a case which comes to my mind just now, as illustrating very forcibly the idea I wish to advance. A lady sixty years old, mother of several grown children; menopause twelve or fifteen years ago; general health had for several years been failing. She was now suffering with considerable backache, pains in lower part of abdomen and pelvis; indeed, the usual train of nervous symptoms and general malaise, loss of appetite and flesh, etc., which usually accompany a considerable uterine and ovarian disturbance. It was the belief that she had some womb trouble which prompted her to apply for medical advice. To the touch the uterus and appendages seemed to be normal for one of her age. The uterus was somewhat atrophied, about the normal mobility, and with little or no sensitiveness. There was no reason for examining the urethra or bladder by the touch. As she did not make any complaint in this direction, this was not done. I placed her in

Sims' position and introduced a Munde-Sims speculum, and, as is my custom, I cast my eyes first upon the meatus, and here I found to my surprise a chronic inflammatory condition of the meatus with a considerable area around it in the same condition.

There was some discharge of a muco-purulent character, and on account of the presence of the neoplasm the caliber of the urethra was considerably lessened. This obstruction to the flow of urine had caused a dilatation of the canal back of it, and hence bagging down of the uretha amounting almost to a urethrocele. This seems pretty clearly a case with a long train of reflex neuroses traceable to the condition of the urethra; and yet so far as the patient knew, she was in utter ignorance of the existence of any local trouble, there being no sensitiveness of the part sufficiently marked to direct her attention to it, and she was surprised when I informed her of its existence. It perhaps came on so gradually, and so insidiously in the beginning as not to set up anything like an acute inflammatory condition, and when the irritation did supervene, the nuerotic irritation was reflected, and this brought on the general malaise and ill-health which we found existing.

If a contracted meatus in the male is capable of producing so much neurotic disturbance, while the patient is still in ignorance of any local irritation, why should not the same cause produce the same effect in the female? Varying of course in intensity and degree, in proportion as the male urethra is more complicated in its construction and anatomical relations, than the female.

Pressure on the urethra and the introduction of the urethral speculum did cause this patient some pain, just as the introduction of the bougie into the male urethra when it reaches the point of irritation gives pain. But the point I wish to emphasize, and the one to which I do not remember ever to have seen reference made, is the possibility of the existence of a local trouble in the female urethra of sufficient consequence to superinduce all these neurotic symptoms, and general ill-health, and yet the patient would give absolutely no history to lead us in this direction in our investigations of the case.

In his fine work on Diseases of Women, Skene mentions the condyloma as "a growth of low grade" and "painless." He also says on page 840, that "The pain in any of these new growths is not always *confined* (italics mine) to the urethra, but may be felt in the back, hips, suprapubic region, thighs, knees and feet." But he does not mention in his well written and elaborate treatises on the bladder and urethra, the possible existence of the conditions and results as I have just described them, without the existence of local symptoms.

DISCUSSION OF DR. K. P. MOORE'S PAPER.

Dr. J. G. Earnest, the first gentleman appointed to discuss this paper, said that he had been greatly interested in the paper, and that he fully endorsed the views of the writer; he especially emphasized the *thorough* excision of these new growths, as had been recommended in the paper read. He did not doubt but that the general practitioner was too apt to overlook these obscure local troubles, and that treatment was often directed to the bladder, and sometimes the womb, when, in reality, the vesical irritation was merely a result of the existence of some local trouble in the urethra. He thought the paper presented by Dr. Moore was timely and pertinent, and would be fruitful of good.

Dr. Virgil O. Hardon next followed in the discussion, and said he could fully endorse the views expressed by the author of the paper, and thought with Dr. Moore and Dr. Earnest that the excision of urethral caruncle should be thorough and complete, yet he would drop a word of caution against the destruction of too much mucus and sub-mucus tissue, for fear of leaving a cicatrix, and thus producing a diminution of the urethral calibre, which, he thought would be a worse condition than the existence of a caruncle. He would also caution against too much dilatation of the urethra for fear of paralyzing this organ, and in this way set up an incontinence, a condition much to be deprecated. He thought that these urethral troubles were rather more over-estimated than under-estimated, and that we frequently were disposed to charge up to the urethra and bladder troubles which were located at the uterus and ovaries ; especially was this the case during the catamenial period. He had often found reflex symptoms of very urgent character referred to the genito-urinary organs, when these organs were in a healthy condition. He referred to one remedy which had been merely touched upon by the author of the paper, as one promising great good in the treatment of a great many urethral and vesical diseases, viz.: Emmett's button-hole operation. He thought that it possessed a simplicity in its execution, and gave relief of a far more decided value than was generally accorded to it. The operation, he was sure, could often be done and a cure effected without the patient even knowing that she possessed a new or artificial meatus. And he looked upon this operation as a means of cure in many of these troubles, superior to dilatation or cauterization.

